# Joint Hypermobility Links Neurodivergence to Dysautonomia and Pain

**DOI:** 10.3389/fpsyt.2021.786916

**Published:** 2022-02-02

**Authors:** Jenny L. L. Csecs, Valeria Iodice, Charlotte L. Rae, Alice Brooke, Rebecca Simmons, Lisa Quadt, Georgia K. Savage, Nicholas G. Dowell, Fenella Prowse, Kristy Themelis, Christopher J. Mathias, Hugo D. Critchley, Jessica A. Eccles

**Affiliations:** ^1^Department of Neuroscience, Brighton and Sussex Medical School, University of Sussex, Brighton, United Kingdom; ^2^Research and Development, Sussex Partnership NHS Foundation Trust, Sussex, United Kingdom; ^3^Autonomic Unit, National Hospital for Neurology and Neurosurgery, London, United Kingdom; ^4^Faculty of Brain Sciences, UCL Queen Square Institute of Neurology, University College London, London, United Kingdom; ^5^School of Psychology, University of Sussex, Brighton, United Kingdom; ^6^Neurodevelopmental Service, Sussex Partnership NHS Foundation Trust, Sussex, United Kingdom; ^7^Centre for Regenerative Medicine and Devices, University of Brighton, Brighton, United Kingdom; ^8^Department of Medicine, Guy's and St Thomas' NHS Foundation Trust, London, United Kingdom; ^9^Department of Psychology, University of Warwick, Coventry, United Kingdom; ^10^Pickering Unit, Neurovascular Medicine, St Mary's Hospital, Imperial College London, London, United Kingdom

**Keywords:** autism, attention deficit hyperactivity disorder (ADHD), autonomic dysfunction, joint hypermobility, Ehlers-Danlos syndrome, neurodevelopmental conditions, pain, Tourette syndrome

## Abstract

**Objectives:**

Autism, attention deficit hyperactivity disorder (ADHD), and tic disorder (Tourette syndrome; TS) are neurodevelopmental conditions that frequently co-occur and impact psychological, social, and emotional processes. Increased likelihood of chronic physical symptoms, including fatigue and pain, are also recognized. The expression of joint hypermobility, reflecting a constitutional variant in connective tissue, predicts susceptibility to psychological symptoms alongside recognized physical symptoms. Here, we tested for increased prevalence of joint hypermobility, autonomic dysfunction, and musculoskeletal symptoms in 109 adults with neurodevelopmental condition diagnoses.

**Methods:**

Rates of generalized joint hypermobility (GJH, henceforth hypermobility) in adults with a formal diagnosis of neurodevelopmental conditions (henceforth neurodivergent group, *n* = 109) were compared to those in the general population in UK. Levels of orthostatic intolerance and musculoskeletal symptoms were compared to a separate comparison group (*n* = 57). Age specific cut-offs for GJH were possible to determine in the neurodivergent and comparison group only.

**Results:**

The neurodivergent group manifested elevated prevalence of hypermobility (51%) compared to the general population rate of 20% and a comparison population (17.5%). Using a more stringent age specific cut-off, in the neurodivergent group this prevalence was 28.4%, more than double than the comparison group (12.5%). Odds ratio for presence of hypermobility in neurodivergent group, compared to the general population was 4.51 (95% CI 2.17–9.37), with greater odds in females than males. Using age specific cut-off, the odds ratio for GJH in neurodivergent group, compared to the comparison group, was 2.84 (95% CI 1.16–6.94). Neurodivergent participants reported significantly more symptoms of orthostatic intolerance and musculoskeletal skeletal pain than the comparison group. The number of hypermobile joints was found to mediate the relationship between neurodivergence and symptoms of both dysautonomia and pain.

**Conclusions:**

In neurodivergent adults, there is a strong link between the expression of joint hypermobility, dysautonomia, and pain, more so than in the comparison group. Moreover, joint hypermobility mediates the link between neurodivergence and symptoms of dysautonomia and pain. Increased awareness and understanding of this association may enhance the management of core symptoms and allied difficulties in neurodivergent people, including co-occurring physical symptoms, and guide service delivery in the future.

## Introduction

Autism spectrum disorder (DSM-V, henceforth “autism”) refers to conditions of neurodevelopmental origin, typically entailing differences in social and emotional interaction, perception (with hypo- and hypersensitivities), and behavior (often having focused interests and preference for routine). Overlap with other neurodevelopmental conditions is common including up to 66% co-occurrence with attention deficit hyperactivity disorder [ADHD; (1)], and chronic tic conditions [e.g., Tourette syndrome (TS), in which there is 60% overlap with other neurodevelopmental conditions (2)]. In this paper, we have intended to align our language with the neurodiversity movement (3), for example using Identity-first description (e.g., “autistic” person rather than “person with autism”) and aimed to avoid using ableist language (4). In addition to the defining neurodevelopmental features, autism and related conditions are recognized to be associated with an increased likelihood of developing physical health problems. These include disorders such as fibromyalgia, irritable bowel syndrome, and fatigue, as well as less clearly defined symptoms related to autonomic dysregulation, notably orthostatic intolerance. An association between neurodevelopmental conditions and joint hypermobility is recognized which might elucidate an increased likelihood of physical symptoms (5).

Joint hypermobility describes the ability of joints to move beyond typically “normal” limits, usually as a consequence of ligamentous laxity, as occurs in connective tissue disorders or other genetic conditions (6). In the general population, joint hypermobility is relatively common, yet prevalence can be difficult to estimate, and this was further complicated historically by a variety of criteria, assessment measures, definitions, and cut-offs used in different hypermobility studies.

One report suggests approximately 20% of the United Kingdom population have joint hypermobility (7); another showed 28% of girls and 11% of boys (among 6,022 children) had generalized joint hypermobility (GJH) (6).

When such hypermobility is associated with other symptoms, typically pain or autonomic dysfunction (6, 8), a diagnosis of hypermobility spectrum disorder [HSD, formerly known as joint hypermobility syndrome (JHS)] or Ehlers-Danlos Syndrome (EDS) may be made. Thirteen types of EDS have been described, with hypermobile EDS (hEDS—previously known as EDS-III or EDS-HT), being the most common (8, 9). 2017 hEDS criteria use an age and sex specific cut-off for GJH (10) in contrast to the GJH major criterion for JHS (11).

Clinical features of HSD and hEDS are not limited to musculoskeletal and cutaneous symptoms (9). Associations exist with cardiovascular autonomic dysfunction (12), gastrointestinal difficulties (13), fatigue (12) and pain syndromes (14), gynecological and obstetric problems (15, 16), and mental health concerns (17–19).

As noted, in addition to these conditions, there is growing recognition that hypermobility is associated with the presence of one or more neurodevelopmental conditions, including autism and ADHD (17–19). Individuals with EDS are reported to be 7.4 times (95% CI: 5.2–10.7) more likely to be autistic than a comparison group (18). Autistic children were shown to have greater mobility of joints (maximum passive joint mobility in degrees of angle were measured for a finger, wrist, elbow, and ankle) compared to a matched comparison group (20). The association between autism and joint hypermobility syndrome/hEDS is further illustrated in a series of case studies (21), highlighting the need for more systematic research for robust characterization of these links (17). ADHD is also associated with GJH: One study reported generalized hypermobility in 32% of 54 patients with ADHD, compared to 14% of a comparison group (22). Another study reported the prevalence of GJH to be 74% in 86 children with ADHD, compared to 13% of a comparison group (23). Moreover, in a population-based matched cohort study in Sweden (*n* = 1,771), individuals with EDS were 5.6 times (95% CI: 4.2–7.4) more likely to have an ADHD diagnosis than those without EDS (18).

Both autism and ADHD are commonly associated with the expression of tics. There has been so far no specific evidence published that links primary developmental tic disorder, exemplified by TS, to joint hypermobility. Nevertheless, the clinical overlap and co-occurrence across neurodevelopmental conditions suggests that people with TS are more likely to manifest joint hypermobility compared to the general population. For example, there are high co-occurrence rates of both ADHD and autism with TS, with estimates ranging from 60 to 80% for people with ADHD and 6.5–50% for autistic people (24). Interestingly, across this neurodevelopmental triad, involvement of fronto-striatal circuitry is implicated in the expression of respective symptomology (25, 26). Co-occurrence aside, even those with an isolated diagnosis of TS may be more predisposed to joint hypermobility than the general population given the links between autonomic dysfunction, premonitory urge sensations, and tics (27, 28).

Orthostatic intolerance is a particular common expression of intermittent cardiovascular autonomic dysfunction in people with hypermobility conditions; symptoms such as light-headedness occur upon standing upright and can be relieved by recumbence (29). Symptoms of orthostatic intolerance can be debilitating (12). Associated syncope, fatigue, and migraines add to clinical burden and reduce quality of life (30).

Orthostatic intolerance is significantly more prevalent in individuals with joint hypermobility or hEDS and is often linked to postural tachycardia syndrome [PoTS; (31)]. Postural tachycardia syndrome is characterized by exaggerated increase in heart rate on standing and is one of the most common manifestations of orthostatic intolerance (32). Orthostatic intolerance was observed in 80% of 35 JHS/EDS-III patients during autonomic testing, half of whom met criteria for postural orthostatic tachycardia syndrome. Furthermore, in a survey of 116 patients diagnosed with JHS/EDS-III, nearly everyone (98%) reported the experience of orthostatic intolerance, experiencing dizziness when getting out of bed in the morning or when exercising in the heat or during/after a hot shower (33). Therefore, orthostatic intolerance is frequent in people with hypermobility.

Given the co-occurrence of hypermobility and neurodevelopmental conditions, it is reasonable to hypothesize that individuals with neurodevelopmental conditions (henceforth described as neurodivergent individuals) are more likely to experience orthostatic intolerance and this association is linked to hypermobility itself. Moreover, a more direct association is proposed between orthostatic hypotension and specific behavioral and emotional differences (34). Correspondingly, in unscreened preschool children, an exaggerated difference in pulse pressure between supine and standing positions predicts higher ADHD symptom scores (35). Moreover, children with this poorer pulse pressure regulation were more likely to be viewed as oppositional and inattentive (35). While this study did not test patients with formally diagnosed ADHD, it points toward a relationship between orthostatic intolerance and ADHD that warrants further scrutiny.

Similarly, there has been a paucity of systematic research characterizing dysautonomia in autistic individuals or people with TS. One case series found five of six autistic patients (aged 12–28) had significant orthostatic intolerance on autonomic function testing (36). Conversely, no clinical signs of dysautonomia during orthostatic challenge (head up tilt) test were observed in 39 autistic boys (37). Clearly more research involving larger samples is required to characterize fully the relationship between neurodevelopmental conditions (including autism and TS) and orthostatic intolerance/dysautonomia.

Pain is a common symptom in individuals with hypermobility (14). Given the observed association between neurodevelopmental conditions and hypermobility, neurodivergent individuals may have an increased likelihood of experiencing pain (38). For instance, an online survey of autistic females found that 100% of those with joint hypermobility (*n* = 85) experienced joint pain, compared to only 29% of those without joint hypermobility [*n* = 20; (39)]. Chronic pain has a detrimental effect on the quality of life of neurodivergent individuals (40), highlighting a need to further characterize these associations within a more representative sample including males (38). In ADHD, where the link to hypermobility related disorders is arguably more established (5), the experience of pain may interact with, and impact negatively on, attention (41). In a study of referrals to a pain clinic, patients with ADHD reported statistically significantly higher mean pain scores compared to patients without an ADHD diagnosis (42). While over three-quarters of neurodivergent (autistic/ADHD) females report chronic pain (40), this study did not test if hypermobility was a mediating factor.

Motivated by the evidence described above of associations between hypermobility, orthostatic intolerance and pain symptoms in neurodivergent individuals, this study examined these factors in combination for the first time, involving an adult sample and including a larger sample of autistic people and people with TS than had been investigated previously. We hypothesized that the neurodivergent group will have higher rates of GJH as compared to a large population cohort study [prevalence of GJH in adolescence in ALSPAC (6)]. We used these data because this cohort represents the most robust indication of prevalence of GJH in the general population so far. This prevalence is similar to those found in a large population survey of adults that assessed GJH by self-report (7). We also explored an age specific criterion for GJH in our neurodivergent and a separate comparison group. This comparison is not possible to make with the published data from the ALSPAC study, requiring a separate comparison group. This study tested whether group differences exist between neurodivergent individuals (specified as those with diagnoses of the following neurodevelopmental conditions, i.e., autism, ADHD, and TS) and a comparison group of individuals without formal diagnoses of neurodevelopmental conditions on measures of GJH, autonomic symptoms, and musculoskeletal symptoms, including pain. We hypothesized that compared to unscreened individuals, a significantly greater proportion of the neurodivergent group will have GJH and experience significantly more symptoms of orthostatic intolerance and musculoskeletal pain. We further hypothesized that more females compared to males would express GJH, as is commonly found, and that greater GJH would predict increased orthostatic intolerance and pain. Finally, we will test whether joint hypermobility mediates the relationship between neurodevelopmental conditions and symptoms of orthostatic intolerance and pain.

## Materials and Methods

### Participants

Data were analyzed from 166 participants, of whom 109 participants had confirmed diagnoses of neurodevelopmental conditions (henceforth neurodivergent group) and 57 participants were in a comparison group. Most of the neurodivergent group (*n* = 87) were assessed in secondary care outpatient clinics in Sussex Partnership NHS Foundation Trust (SPFT; including a specialist Neurodevelopmental Service for adults) as part of a study of prevalence of hypermobility and autonomic symptoms (approved by the South East Coast NRES Ethics Committee; 12.LO.1942) between 2013 and 2016. Clinical notes of patients were evaluated to confirm that they had received a clinical diagnosis of autism, ADHD, or TS. Additional individuals with TS (*n* = 22) were originally recruited to another approved study (South East Coast NRES Ethics Committee; 15.LO.0109).

Eligibility criteria for the adult neurodivergent group included confirmed specialist diagnosis of neurodevelopmental condition (autism, ADHD, TS). All patients at SPFT were assessed at that time using DSM IV criteria and semi-structured interviews relating to the DIVA assessment for ADHD and Royal College of Psychiatrists (England) Interview Guide for the Diagnostic Assessment of Adults with Autism Spectrum Disorder. The TS participants recruited via the Tourette study were recruited predominately via the charity Tourettes Action and had to have a confirmed diagnosis of TS by a named specialist. Co-occurring neurodevelopmental conditions were also noted, and again had to be diagnosed by a named specialist.

These neurodivergent participants were compared to a group of adults with no diagnosed neurodevelopmental, mental health, or neurological conditions. This comprised those who were recruited to a study to validate the autonomic symptoms questionnaire measure [an autonomic and quality of life self-administered questionnaire; AQQoL (43); *n* = 29] and also adults who were recruited for a separate psychophysiological study, which included the same measurements as the validation study (Brighton and Sussex Medical School RGEC; 13/122/CRI). The comparison group were not screened for neurodevelopmental or other conditions.

This study involved patient and public involvement and the authorship includes neurodivergent individuals.

### Measures and Materials

The autonomic symptoms questionnaire measure (AQQoL) (43) was used to quantify subjective experiences of orthostatic intolerance and musculoskeletal symptoms and incorporated the Beighton Scoring System for joint hypermobility (44). The Beighton Scoring System was administered as a clinical examination by a trained clinician/researcher.

Two definitions of GJH were used in this study. Firstly, Beighton scores of 4 and above were classified as indicative of GJH in line with the general population data previously published (6) and the now superseded major criterion for Joint Hypermobility Syndrome (11) (henceforth JHS GJH). Given controversies surrounding categorization of generalized joint hypermbolity and change to diagnostic criteria, additionally age specific cut-offs of Beighton Scoring System were used in line with Criterion 1 of the 2017 hEDS diagnostic criteria, whereby pubertal men and women up to the age of 50 were considered positive with a Beighton score ≥5, and men and women over the age of 50 were considered positive with a Beighton score of ≥4 (10). This will be named hEDS GJH henceforth.

Participants were asked to indicate on a Five-point Likert scale (from “no” to “yes–daily”) how often they experienced a set of symptoms associated with orthostatic intolerance (e.g., “do you feel dizzy or lightheaded?”). Occurrence of musculoskeletal symptoms (predominately pain) were also surveyed [e.g., “do you have any of the following symptoms? (a) pains in the knees; (b) pains in the fingers”]. Participants were asked to indicate musculoskeletal symptom frequency on a three-point Likert scale [from “no” to “yes—for longer than 3 months”]. Most participants completed this full AQQoL questionnaire, except the 22 patient participants in the TS study who only completed the Beighton Scoring and orthostatic intolerance subscale of the AQQoL.

### Data Preparation for Statistical Analyses

Data from the ALSPAC birth cohort (6) were used to compute the odds ratio of having GJH in the neurodivergent group compared to the general population. In this birth cohort, the Beighton score was available for 6,022 adolescents from the general population. While these data were collected in adolescents, the levels of GJH in this sample is comparable to that shown in adults (18% of the population) (7)—together these findings represent the largest prevalence studies of GJH in a general population setting.

In the original validation of the AQQoL, the Beighton score was included in the total musculoskeletal subscale. Here, the Beighton score was analyzed separately as it was hypothesized that there would be a difference in Beighton scores between neurodivergent and the comparison group.

Binary logistic regression was used to calculate the odds ratios of having GJH for patients compared to the population-level comparison group (ALSPAC birth cohort). Binary logistic regression was used to test for group differences between the neurodivergent group and the comparison group in the proportion of people with GJH. Separate predictor models used sex as a covariate.

Mann-Whitney U-tests were used to test for group differences between neurodivergent group and the comparison group, comparing: (1) age, (2) Beighton scores, (3) orthostatic intolerance symptom scores, and (4) musculoskeletal scores. This approach was used because scores on these four measures for each group were not normally distributed. Spearman's correlations were also calculated between Beighton scores and: (1) orthostatic intolerance symptom scores, and (2) musculoskeletal scores given the predicted relationships between these measures (i.e., higher Beighton scores will be positively correlated with increased orthostatic intolerance symptom scores and musculoskeletal scores). Rain cloud plots were used to visualize data differences between groups where appropriate (45) and generated in R (46).

To explore potential mechanistic associations between neurodivergence, joint hypermobility and symptoms, mediation analyses were performed. As such, to investigate whether Beighton score mediated the relationship between neurodivergence and assessed symptoms (orthostatic intolerance symptoms and musculoskeletal symptoms), an estimation of indirect effects was performed using PROCESS macro v3.5 for SPSS by Hayes (47). The 95% bootstrapped confidence interval for the indirect effect is based on 1,000 samples and considered significant if the bootstrapped confidence intervals do not cross zero. For these analyses the predictor variable was neurodivergence, the mediator variable was Beighton Score, and the outcome variables were orthostatic intolerance symptoms score and musculoskeletal symptoms score.

## Results

For full description of the groups see Table 1. In the neurodivergent group 67 were assigned male at birth (62%) and 42 assigned female at birth (38%), and in the comparison group 26 assigned male at birth (46%) and 31 (54%) assigned female at birth. This was a statistically significant difference (*p* = 0.051). The mean age of the neurodivergent group was 34.9 years (*SD* = 11.3), and the mean age of the comparison group was 39.2 years (*SD* = 14.95). There was no significant difference in ages between the groups.

**Table 1:** Group characteristics.

**Characteristics**	**Neurodevelopmental condition**	**Comparison group**	**Difference in demographics**
	**participants (*n* = 109)**	**(*n* = 57)**	**between groups**
Age in years (*M, SD*)	34.9 (11.3) Range: 18–61	39.2 (14.95) Range: 18–68	*p* = 0.1.01
Sex, male (% of group)	67 (62%)	26(46%)	*p* = 0.051
Sex, female (% of group)	42 (38%)	31 (54%)	
Beighton score/9 (*M, SD*)	3.2 (2.6)	1.42 (2.3)	*p* ≤ 0.001
Orthostatic intolerance symptom score/120 (*M, SD*)	24.2 (15.6)	5.1 (4.3)	*p* ≤ 0.001
Musculoskeletal score/14 (*M, SD*)	6.8 (3.7)	3.58 (2.9)	*p* ≤ 0.001

### Neurodevelopmental and Mental Health Diagnoses

Twenty-seven patients were diagnosed autistic (25% of patients). Fifty-six of the patients were diagnosed with ADHD (51% of patients), and 15 patients had a diagnosis of TS (14% of patients). Two patients had co-occurring diagnoses of autism and ADHD (2% of patients) and eight patients were diagnosed with ADHD and TS (7% of patients). One patient had a diagnosis of autism, ADHD and TS (<1% of patients).

### Generalized Joint Hypermobility

Beighton score was significantly higher in the neurodivergent group (*M* = 3.2, *SD* = 2.6) than in the comparison group (*M* = 1.42, *SD* = 2.3; *U* = 4,434, *z* = 4.62, *p* <0.001; Table 1; **Figure 3**).

#### Beighton Score ≥4 (JHS GJH)

**Figure 1:**
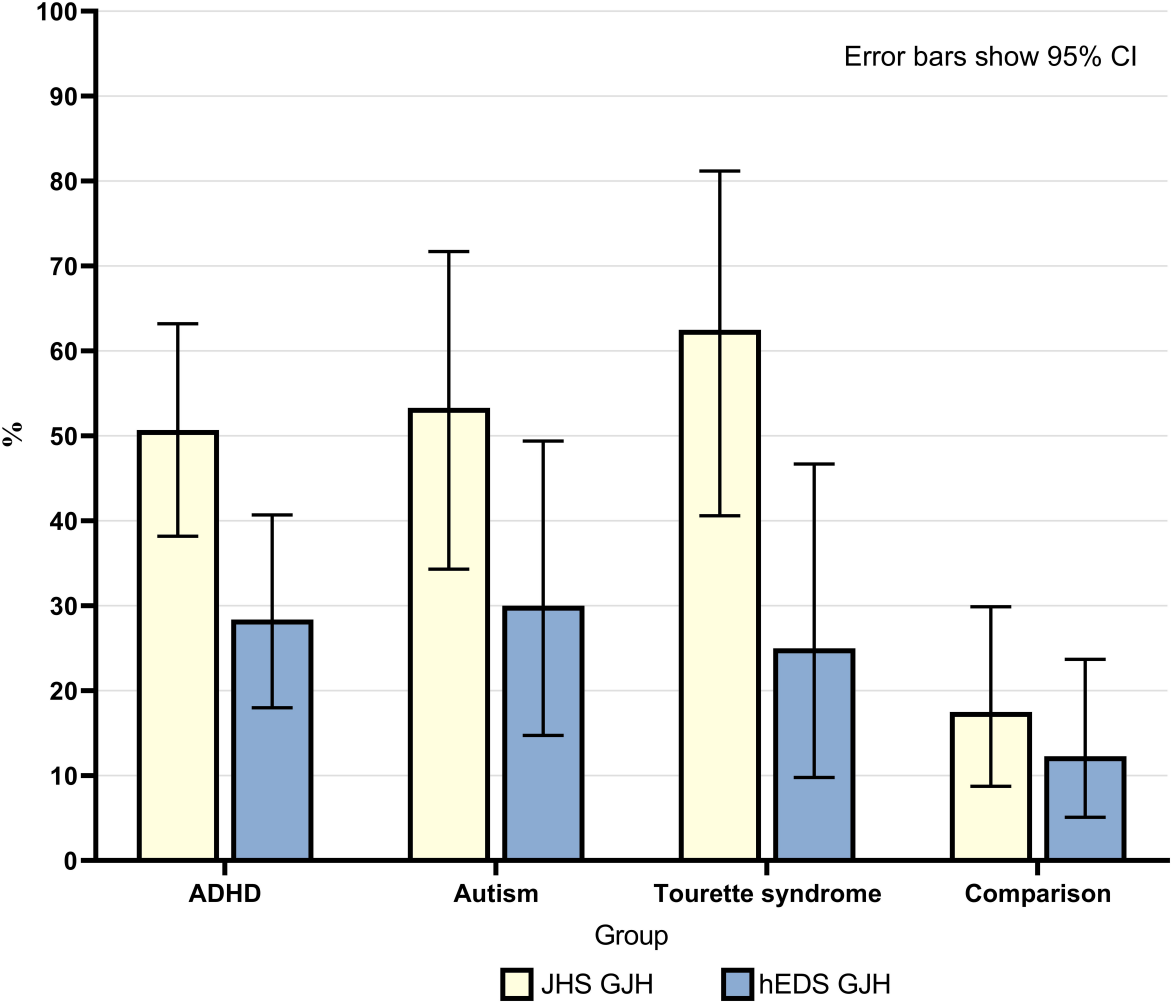
Percentage of individuals in each group who had generalized joint hypermobility according to both JHS criteria (Beighton score ≥4) and 2017 hEDS criteria (age specific cut-off). Error bars show 95% CI.

**Figure 2:**
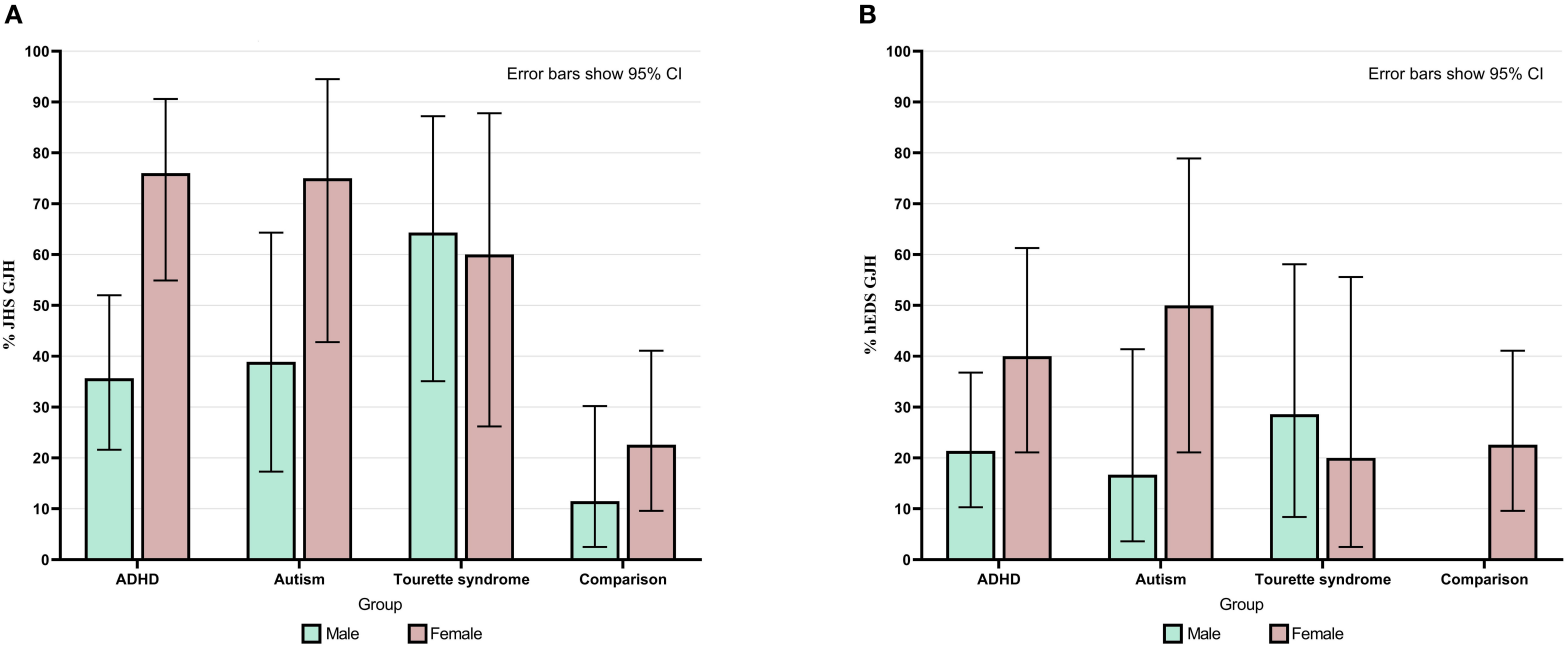
**(A)** Percentage of individuals in each group and within each sex who had generalized joint hypermobility according to JHS criteria (Beighton score ≥4). **(B)** Percentage of individuals in each group and within each sex who had generalized joint hypermobility according to 2017 hEDS criteria (age specific cut-off). Error bars show 95% CI.

Overall, 50% of neurodivergent individuals met criteria for GJH compared to 17.5% in the comparison group (see Figure 1). There were marked sex differences; the prevalence in the neurodivergent females assigned at birth was 69% compared to 22.6% in the comparison group. See Figure 2 for a breakdown per neurodivergent condition. The presence of GJH significantly predicted membership of the neurodivergent group (OR 4.79, 95% CI 2.20–10.43, *p* < 0.001). This relationship remained after adjusting for sex (OR 6.45, 95% CI 2.79–14.92, *p* < 0.001).

Generalized joint hypermobility was 4.51 (95% CI 2.17–9.37) times higher if individuals were autistic, 4.34 (95% CI 2.67–7.03) times higher if individuals had an ADHD diagnosis and 7.02 (95% CI 3.06–16.1) times higher if individuals had a diagnosis of TS, compared to the general population sample.

Across all participants, there was a significant association between sex and whether individuals had GJH: Females were more likely to have GJH [$\chi_{\left(1\right)}^{2}$ = 5.64, *p* = 0.018]. There was a significant interaction of group membership on this relationship [*F*_(2)_ = 6.23, *p* = 0.002]; i.e., the strength of the relationship between sex and GJH was significantly greater in the neurodivergent group.

#### Age Specific Beighton Cut-Off (HEDS GJH)

Overall, 28.4% of neurodivergent individuals met criteria for the more stringent age specific GJH criterion compared to 12.3% in the comparison group (see Figure 1). There were marked sex differences; the prevalence in neurodivergent females assigned at birth was 40.5% compared to 22.6% in the comparison group. See Figure 2 for a breakdown per neurodivergent condition. The presence of age specific GJH significantly predicted membership of the neurodivergent group (OR 2.84, 95% CI 1.16–6.94, *p* = 0.022). This relationship remained after adjusting for sex (OR 3.68, 95% CI 1.44–9.36, *p* = 0.006).

Again, there was a significant association between sex and GJH using the age specific cut-off [$\chi_{\left(1\right)}^{2}$ = 6.39, *p* = 0.007). Again, this relationship is significantly stronger in the neurodivergent group, i.e., there is a formal interaction of group membership on the relationship between GJH and sex [*F*_(2)_ = 5.20, *p* = 0.006].

### Orthostatic Intolerance

**Figure 3:**
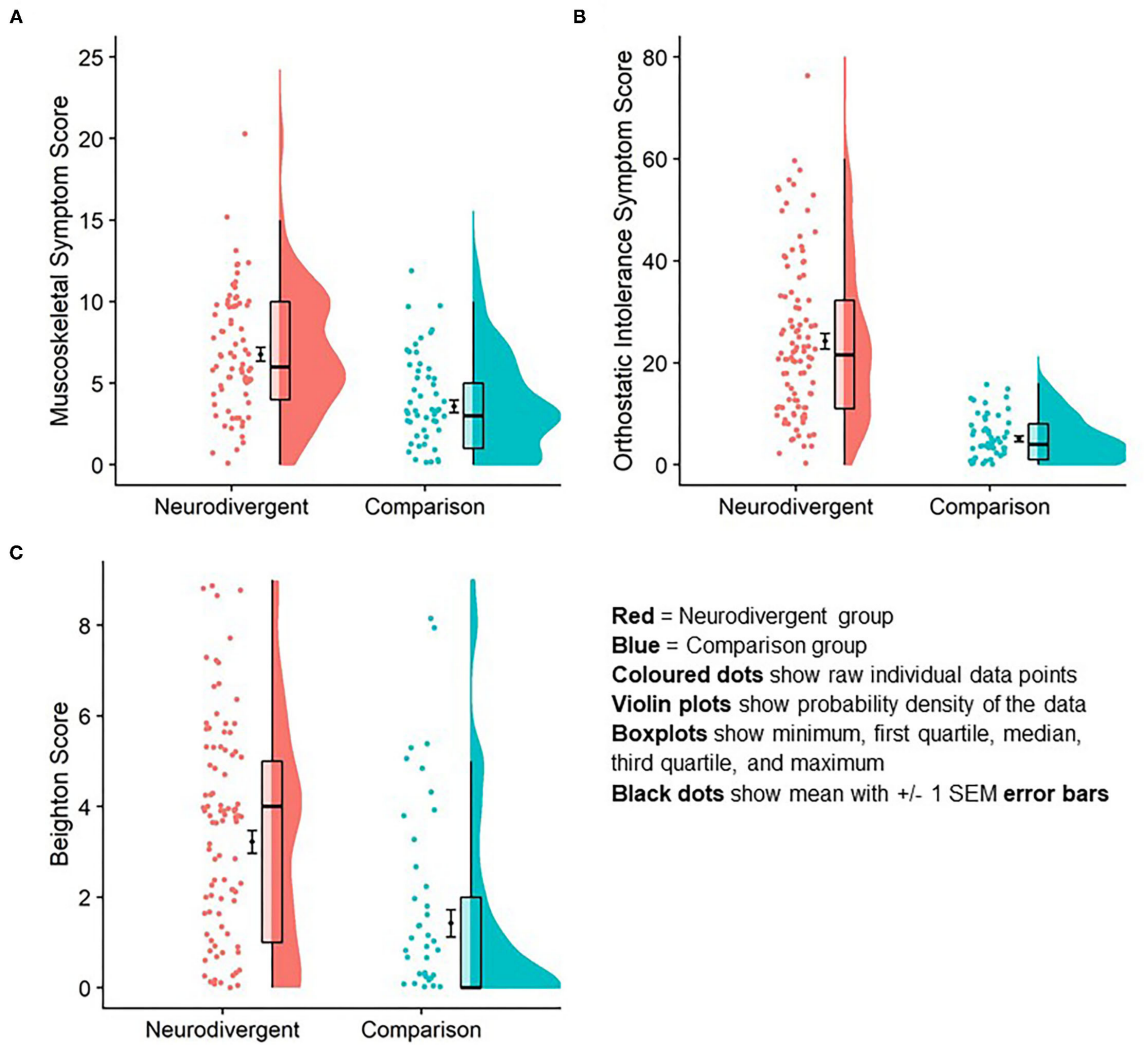
Difference in orthostatic intolerance symptom score **(A)**; musculoskeletal symptom score **(B)** and Beighton score **(C)** between neurodivergent and comparison group. Graphic is a data rain cloud illustrating raw data, median and interquartile range, and probability density for each variable in each group. Mean and standard error are visualized also.

Mean orthostatic intolerance symptom score in neurodivergent participants was significantly higher (*M* = 24.2, *SD* = 15.6) compared to individuals in the comparison group (*M* = 5.1, *SD* = 4.3; *U* = 5,441, *z* = 8.76, *p* < 0.001; Figure 3).

**Figure 4:**
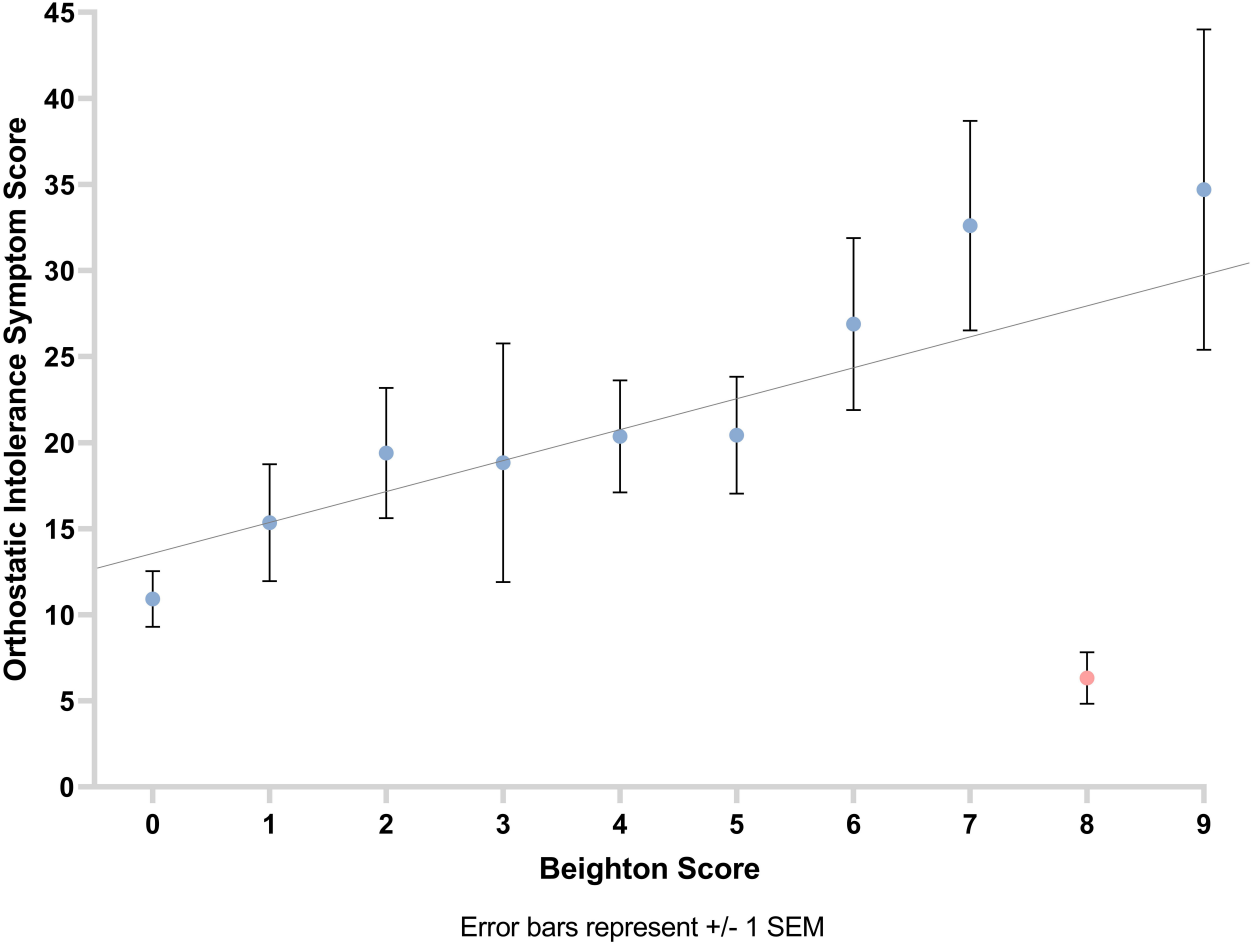
Graph showing the relationship between orthostatic intolerance symptom score and Beighton score with line of best fit. Error bars show ±1 standard error of the mean.

We observed a positive correlation between orthostatic intolerance symptom score and GJH (*r*_*s*_ =‘0.39, *p* < 0.001). This suggests that the higher the participants' Beighton score is, the greater their experience of symptoms of orthostatic intolerance (Figure 4).

### Musculoskeletal Pain

Mean musculoskeletal symptom score was significantly higher in neurodivergent participants (*M* = 6.7 *SD* = 3.7) compared to individuals in the comparison group (*M* = 3.58, *SD* = 2.93; *U* = 1811.5, *z* = 4.95, *r* = 0.48, *p* < 0.001; Figure 3).

**Figure 5:**
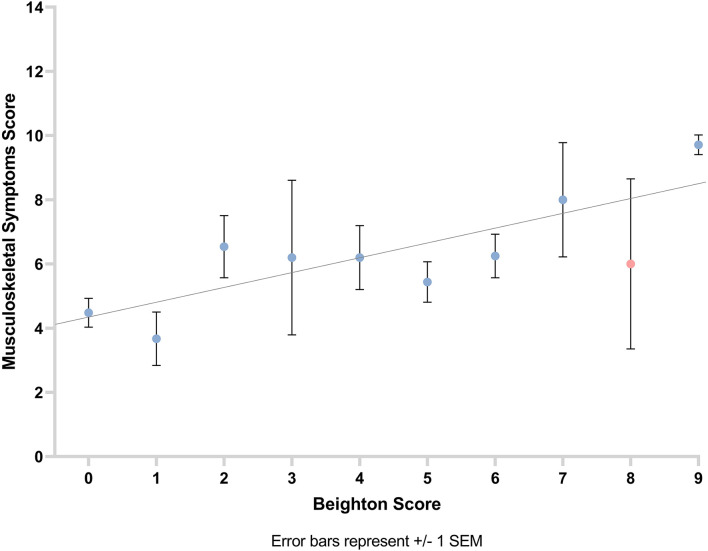
Graph showing the relationship between musculoskeletal symptom score and Beighton score with line of best fit. Error bars show ±1 standard error of the mean.

A positive correlation was observed between musculoskeletal symptom score and Beighton score (number of hypermobile joints as per Beighton score; *r*_*s*_ = 0.28, *p* = 0.001). This suggests that the greater the participants' Beighton score, the higher their musculoskeletal symptom score (Figure 5).

### Mediation Analyses

**Figure 6:**
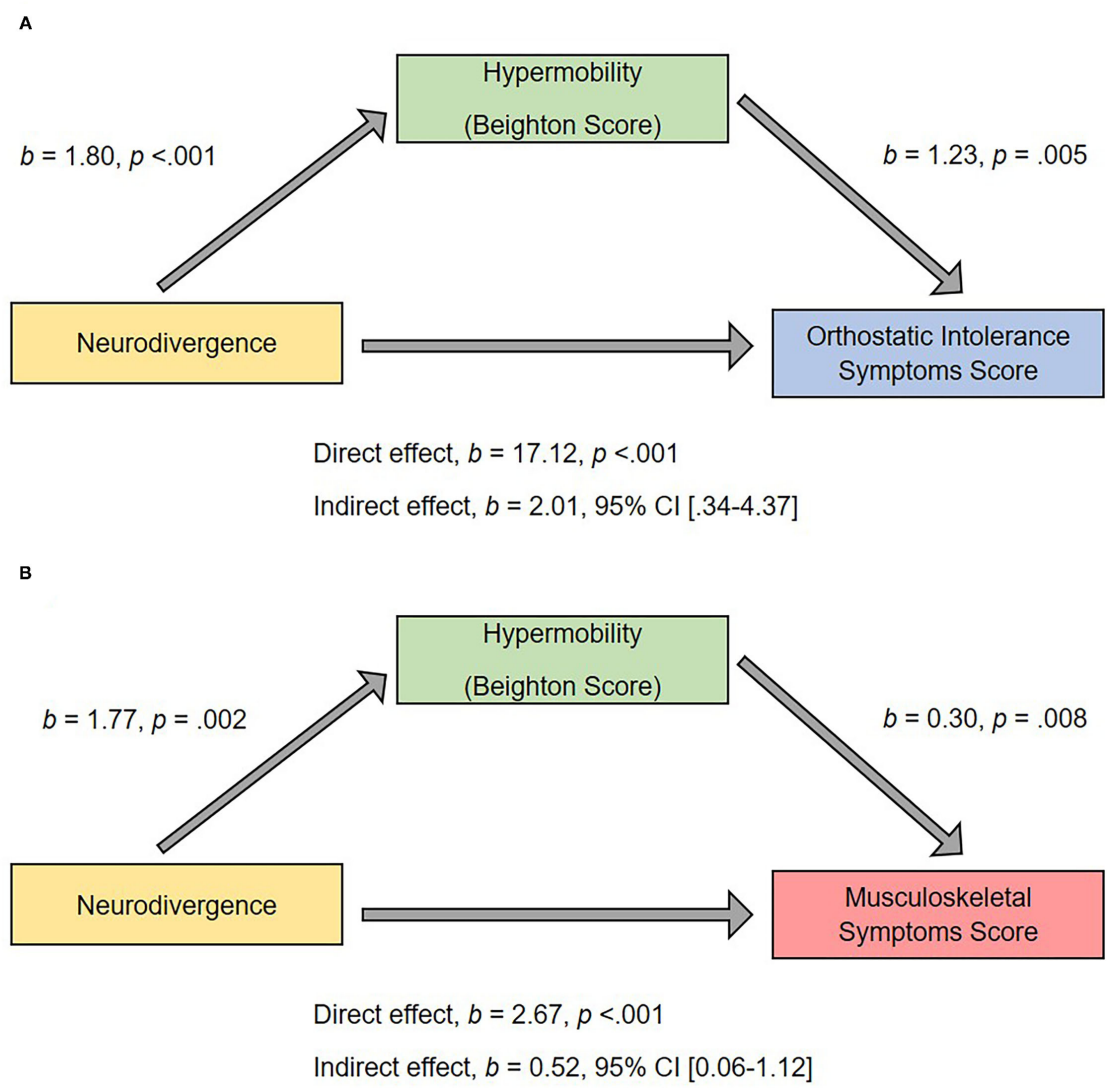
Hypermobility as a mediator of predictor relationship between neurodivergent status and **(A)** orthostatic intolerance score; **(B)** musculoskeletal score. The confidence interval for the indirect effect is a bootstrapped confidence interval based on 1,000 samples.

There was a significant indirect effect of neurodivergence on orthostatic intolerance symptoms through Beighton score (*b* = 2.01, 95% CI 0.34–4.37), and a significant indirect effect of neurodivergence on musculoskeletal symptoms through Beighton score (*b* = 0.52, 95% CI 0.06–1.12; Figure 6), suggesting that hypermobility is mediating the association (i.e., the direct effect) between membership of the neurodivergent group on greater physical symptoms.

## Discussion

This is the first study to investigate the prevalence of GJH, orthostatic intolerance symptoms, and pain (in the form of musculoskeletal symptoms) across neurodivergent adults (including those diagnosed with TS) in contrast to a comparison group, and also to present associated prevalence data on two different definitions of GJH. All hypotheses were supported: Neurodivergent individuals were significantly more likely to have GJH, and to experience orthostatic intolerance and musculoskeletal symptoms. Generalized joint hypermobility was strikingly more common in neurodevelopmental females and was associated both with symptoms of orthostatic intolerance and pain. Moreover, the relationship between neurodivergence and co-occuring physical symptoms was mediated by hypermobility, providing a potential mechanistic link between neurodivergence and physical symptoms. It may be that constitutional variation in connective tissue is a unifying feature in the predisposition to neurodivergence, pain, and symptoms of dysautonomia.

Regardless of specific criterion used, prevalence of GJH was more than double in neurodivergent groups, confirming a hypothesized association that was suggested by earlier brain imaging findings linking hypermobility to possible neural correlates of neurodivergence (48). The over-representation of hypermobility in autistic participants also extends a growing literature from case studies, children, and cohort research (18, 20, 21). Fifty-one percent of individuals diagnosed with ADHD had GJH (according to the older, broader cut-off), which falls between the lower estimate in previous research with adults [32% of 54 patients (22)] and the higher estimate in research with children [74% of 86 patients (23)] with ADHD. Interestingly, an animal model provides cross-species evidence of the association between hypermobility and core features of ADHD (49). However, the co-occurrence of these conditions makes it difficult to tease them apart, especially where the co-occurrence between autism and ADHD is high (1). We also observed high rates of GJH in people with TS and provide the first reported systematic estimate of this association. Overall, our findings highlight the increased prevalence of GJH in neurodivergence.

The significantly higher prevalence of orthostatic intolerance symptoms experienced by neurodivergent individuals compared to a comparison group provides direct evidence for this suggestion within more anecdotal literature (34, 36). Moreover, our larger study used quantitative data from patients a neurodivergent group, extending an association shown between orthostatic symptoms and ADHD questionnaire score in a smaller sample of individuals who did not have a formal diagnosis of this or another neurodevelopmental condition (35). Our mediation analysis suggests that hypermobility is a mediating factor in the relationship between neurodivergence and such symptoms.

There may also be a neural contribution to the higher prevalence of orthostatic intolerance symptoms in neurodivergence. The insular cortex underpins interoception; the process by which we perceive physiological information concerning the current state of the body (50). As well as receiving afferent information from bodily systems, interoceptive networks contribute to efferent autonomic adaptations, in pursuit of homeostasis (51). Altered insular function in neurodivergence may foster noisier integration of interoceptive signals (52), leading to larger orthostatic intolerance symptoms. Interestingly, participants with hypermobility show insular hyperactivation to affective stimuli during fMRI (53), and structural and functional alterations to insular cortex are also seen in neuroimaging studies of neurodivergence [e.g., TS (54, 55)].

Previous work suggests that neurodivergent people might experience more symptoms of pain compared to people without a neurodevelopmental condition diagnosis [e.g., (42)]. Our mediation analysis directly links hypermobility as a mediator of a relationship between neurodivergence and pain. However, one possible limitation of this study concerns the measurement of pain. Using the AQQoL as a self-report measure for musculoskeletal symptoms (therefore pain) may have under-estimated participants' experience of pain as it does not ask questions about all areas of the body (such as the neck). Alternatively, using a more widely validated measure of pain could have been useful. For example, the Brief Pain Inventory–Short Form (56), would allow more parts of the body to be considered. However, even then, additional work would be needed ensure reliable categorization of where participants indicated the location of felt pain. Another option would be to use the Pain Interference Index (57) to assess the subsequent impact of pain (irrespective of where it occurred in the body) on functioning. This scale has been validated for use in adults and could be beneficial to use in future research. There is a shift toward considering the impact of pain on daily life, rather than focusing on pain frequency, because of the possible clinical implications.

A further limitation concerns the measurement of GJH as participants were not assessed as to whether they met diagnostic criteria for HSD or hEDS. There is considerable debate about categorization and measurement of GJH (58). Further work should focus on symptomatic hypermobility in neurodivergence as it is likely that these individuals will need more targeted interventions, as people with HSD/hEDS are more likely to experience pain and other physical symptoms which can negatively impact on quality of life. Furthermore, we only assessed symptoms of orthostatic intolerance, rather than assessing autonomic function directly, and future work should incorporate autonomic function testing, as symptoms of orthostatic intolerance may overlap with symptoms of other processes.

This study provides compelling evidence for increased prevalence of joint hypermobility—a constitutional variant in systemic connective tissue—in neurodivergent individuals and posits a mediating mechanism through which physical symptoms are linked to neurodivergence, via such variation in connective tissue. Autonomic cardiovascular dysregulation (orthostatic intolerance) and musculoskeletal pain were also expressed at higher rates across neurodivergent individuals and showed a clear relationship to joint hypermobility. Differences in connective tissue may be a mediating factor for both these associations, causing pooling of blood in lax peripheral vessels [as in some forms of PoTS (59)] and enhancing sensitivity to tissue stretch and damage. Here, we highlight the relevance in the context of a set of neurodevelopmental conditions where differences in perceptual sensitivity, including abnormalities in proprioception and interoception (27, 60), can contribute to psychosocial difficulties associated with these conditions and also to the (often under recognized) increased likelihood of chronic physical symptoms that further decrease quality of life. Autism, ADHD, and developmental tic disorder (TS) often share complex symptoms, consistent with overlapping neurodevelopmental etiopathology. Increasingly, services need to recognize such complexity and move beyond “exclusive” diagnostic categories and traditional boundaries between physical and mental health (61). This paper sets the scene for larger studies and will also facilitate targeting of interventions to enhance quality of life across psychological and physical domains in neurodivergence.

## Data Availability Statement

The raw data supporting the conclusions of this article will be made available by the authors, without undue reservation.

## Ethics Statement

The studies involving human participants were reviewed and approved by South East Coast NRES Ethics Committee; 12.LO.1942 South East Coast NRES Ethics Committee; 15.LO.0109; Brighton and Sussex Medical School RGEC; 13/122/CRI. The participants provided their written informed consent to participate in this study.

## Author Contributions

JE and HC designed the study with input from VI and CM. JE, JC, VI, KT, and LQ analyzed data. LQ and GS visualized data. JE, JC, and VI drafted the manuscript. All authors contributed to the finalized manuscript.

## Funding

Funding for this particular project came via a fellowship to JE (MRC MR/K002643/1). JE is currently supported by an MQ Versus Arthritis Fellowship (MQF 17/19).

## Conflict of Interest

The authors declare that the research was conducted in the absence of any commercial or financial relationships that could be construed as a potential conflict of interest. The handling editor CB-V declared a past co-authorship/collaboration with one of the authors HC.

## Publisher's Note

All claims expressed in this article are solely those of the authors and do not necessarily represent those of their affiliated organizations, or those of the publisher, the editors and the reviewers. Any product that may be evaluated in this article, or claim that may be made by its manufacturer, is not guaranteed or endorsed by the publisher.

## Supplementary Material

Supplementary Table 1 Recorded diagnoses in neurodivergent participants.

## References

[1] Davis NO Kollins SH. Treatment for co-occurring attention deficit/hyperactivity disorder and autism spectrum disorder. Neurotherapeutics. (2012) 9:518–30. 10.1007/s13311-012-0126-9 22678458 PMC3441928

[2] Huisman-Van Dijk HM Schoot R Rijkeboer MM Mathews CA Cath DC. The relationship between tics, OC, ADHD and autism symptoms: a cross-disorder symptom analysis in Gilles de la Tourette syndrome patients and family-members. Psychiatry Res. (2016) 237:138–46. 10.1016/j.psychres.2016.01.051 26826899 PMC5137472

[3] Kapp SK Gillespie-Lynch K Sherman LE Hutman T. Deficit, difference, or both? Autism and neurodiversity. Dev Psychol. (2013) 49:59–71. 10.1037/a0028353 22545843

[4] Bottema-Beutel K Kapp SK Lester JN Sasson NJ Hand BN. Avoiding ableist language: suggestions for autism researchers. Autism Adulthood. (2021) 3:18–29. 10.1089/aut.2020.0014 PMC8992888 36601265

[5] Baeza-Velasco C Sinibaldi L Castori M. Attention-deficit/hyperactivity disorder, joint hypermobility-related disorders and pain: expanding body-mind connections to the developmental age. ADHD Attent Defic Hyperact Disord. (2018) 10:163–75. 10.1007/s12402-018-0252-2 29446032

[6] Clinch J Deere K Sayers A Palmer S Riddoch C Tobias JH. Epidemiology of generalized joint laxity (hypermobility) in fourteen-year-old children from the UK: a population-based evaluation. Arthritis Rheum. (2011) 63:2819–27. 10.1002/art.30435 21547894 PMC3164233

[7] Mulvey MR Macfarlane GJ Beasley M Symmons DP Lovell K Keeley P. Modest association of joint hypermobility with disabling and limiting musculoskeletal pain: results from a large-scale general population-based survey. Arthritis Care Res (Hoboken). (2013) 65:1325–33. 10.1002/acr.21979 23401475

[8] Sobey G. Ehlers–Danlos syndrome: how to diagnose and when to perform genetic tests. Arch Dis Child. (2015) 100:57–61. 10.1136/archdischild-2013-304822 24994860

[9] Demmler JC Atkinson MD Reinhold EJ Choy E Lyons RA Brophy ST. Diagnosed prevalence of Ehlers-Danlos syndrome and hypermobility spectrum disorder in Wales, UK: a national electronic cohort study and case–control comparison. BMJ Open. (2019) 9:e031365. 10.1136/bmjopen-2019-031365 31685485 PMC6858200

[10] Malfait F Francomano C Byers P Belmont J Berglund B Black J. The 2017 international classification of the Ehlers-Danlos syndromes. Am J Med Genet C Semin Med Genet. (2017) 175:8–26. 10.1002/ajmg.c.31552 28306229

[11] Grahame R Bird HA Child A. The revised (Brighton 1998) criteria for the diagnosis of benign joint hypermobility syndrome (BJHS). J Rheumatol. (2000) 27:1777–9. Available online at: https://pubmed.ncbi.nlm.nih.gov/10914867/10914867

[12] Hakim A O'callaghan C De Wandele I Stiles L Pocinki A Rowe P. Cardiovascular autonomic dysfunction in Ehlers-Danlos syndrome-Hypermobile type. Amer J Med Genet C Semin Med Genet. (2017) 175:168–74. 10.1002/ajmg.c.31543 28160388

[13] Fikree A Chelimsky G Collins H Kovacic K Aziz Q. Gastrointestinal involvement in the Ehlers-Danlos syndromes. Amer J Med Genet C Semin Med Genet. (2017) 175:181–7. 10.1002/ajmg.c.31546 28186368

[14] Chopra P Tinkle B Hamonet C Brock I Gompel A Bulbena A. Pain management in the Ehlers-Danlos syndromes. Amer J Med Genet C Semi Med Genet. (2017) 175:212–9. 10.1002/ajmg.c.31554 28186390

[15] Hugon-Rodin J Lebègue G Becourt S Hamonet C Gompel A. Gynecologic symptoms and the influence on reproductive life in 386 women with hypermobility type Ehlers-Danlos syndrome: a cohort study. Orphanet J Rare Dis. (2016) 11:124. 10.1186/s13023-016-0511-2 27619482 PMC5020453

[16] Pezaro S Pearce G Reinhold E. Hypermobile Ehlers-Danlos Syndrome during pregnancy, birth and beyond. Brit J Midwif. (2018) 26:217–23. 10.12968/bjom.2018.26.4.217

[17] Bulbena A Baeza-Velasco C Bulbena-Cabré A Pailhez G Critchley H Chopra P. Psychiatric and psychological aspects in the Ehlers-Danlos syndromes. Amer J Med Genet C Semin Med Genet. (2017) 175:237–45. 10.1002/ajmg.c.31544 28186381

[18] Cederlöf M Larsson H Lichtenstein P Almqvist C Serlachius E Ludvigsson JF. Nationwide population-based cohort study of psychiatric disorders in individuals with Ehlers–Danlos syndrome or hypermobility syndrome and their siblings. BMC Psychiatry. (2016) 16:207. 10.1186/s12888-016-0922-6 27377649 PMC4932739

[19] Sharp HEC Critchley HD Eccles JA. Connecting brain and body: transdiagnostic relevance of connective tissue variants to neuropsychiatric symptom expression. World J Psychiatry. (2021) 11:805–20. 10.5498/wjp.v11.i10.805 34733643 PMC8546774

[20] Shetreat-Klein M Shinnar S Rapin I. Abnormalities of joint mobility and gait in children with autism spectrum disorders. Brain Dev. (2014) 36:91–6. 10.1016/j.braindev.2012.02.005 22401670

[21] Sinibaldi L Ursini G Castori M. Psychopathological manifestations of joint hypermobility and joint hypermobility syndrome/Ehlers-Danlos syndrome, hypermobility type: the link between connective tissue and psychological distress revised. Amer J Med Genet C Semin Med Genet. (2015) 169:97–106. 10.1002/ajmg.c.31430 25821094

[22] Dogan SK Taner Y Evcik D. Benign joint hypermobility syndrome in patients with attention deficit/hyperactivity disorders. Arch Rheumatol. (2011) 26:187–92. 10.5606/tjr.2011.029

[23] Shiari R Saeidifard F Zahed G. Evaluation of the prevalence of joint laxity in children with attention deficit/hyperactivity disorder. Ann Paediatr Rheumatol. (2013) 2:78. 10.5455/apr.032420131219

[24] Cavanna AE Servo S Monaco F Robertson MM. The behavioral spectrum of Gilles de la Tourette syndrome. J Neuropsychiatry Clin Neurosci. (2009) 21:13–23. 10.1176/jnp.2009.21.1.13 19359447

[25] Rapanelli M Frick LR Pittenger C. The role of interneurons in autism and Tourette Syndrome. Trends Neurosci. (2017) 40:397–407. 10.1016/j.tins.2017.05.004 28578790 PMC5528854

[26] Israelashvili M Yael D Vinner E Belelovsky K Bar-Gad I. Common neuronal mechanisms underlying tics and hyperactivity. Cortex. (2020) 127:231–47. 10.1016/j.cortex.2020.02.010 32244155

[27] Rae CL Larsson DEO Garfinkel SN Critchley HD. Dimensions of interoception predict premonitory urges and tic severity in Tourette syndrome. Psychiatry Res. (2019) 271:469–75. 10.1016/j.psychres.2018.12.036 30544073

[28] Hawksley J Cavanna AE Nagai Y. The role of the autonomic nervous system in Tourette Syndrome. Front Neurosci. (2015) 9:117. 10.3389/fnins.2015.00117 26074752 PMC4444819

[29] Stewart JM. Common syndromes of orthostatic intolerance. Pediatrics. (2013) 131:968–80. 10.1542/peds.2012-2610 23569093 PMC3639459

[30] De Wandele I Calders P Peersman W Rimbaut S De Backer T Malfait F. Autonomic symptom burden in the hypermobility type of Ehlers–Danlos syndrome: a comparative study with two other EDS types, fibromyalgia, and healthy controls. Semin Arthritis Rheum. (2014) 44:353–61. 10.1016/j.semarthrit.2014.05.013 24968706

[31] Roma M Marden CL De Wandele I Francomano CA Rowe PC. Postural tachycardia syndrome and other forms of orthostatic intolerance in Ehlers-Danlos syndrome. Autonomic Neuroscience. (2018) 215:89–96. 10.1016/j.autneu.2018.02.006 29519641

[32] Celletti C Camerota F Castori M Censi F Gioffrè L Calcagnini G. Orthostatic intolerance and postural orthostatic tachycardia syndrome in joint hypermobility syndrome/Ehlers-Danlos syndrome, hypermobility type: neurovegetative dysregulation or autonomic failure? Biomed Res Int. (2017) 2017:1–7. 10.1155/2017/9161865 28286774 PMC5329674

[33] Chan C Krahe A Lee YT Nicholson LL. Prevalence and frequency of self-perceived systemic features in people with joint hypermobility syndrome/Ehlers-Danlos syndrome hypermobility type. Clin Rheumatol. (2019) 38:503–11. 10.1007/s10067-018-4296-7 30232714

[34] Perlmuter LC Sarda G Casavant V Mosnaim AD. A review of the etiology, associated comorbidities, and treatment of orthostatic hypotension. Am J Ther. (2013) 20:279–91. 10.1097/MJT.0b013e31828bfb7f 23656967

[35] Casavant V Chae C Sherwani A Perlmuter LC. Subclinical orthostatic pulse pressure confirms mothers' ratings of ADHD in preschoolers. Psychophysiology. (2012) 49:708–12. 10.1111/j.1469-8986.2012.01351.x 22404137

[36] Goodman B. Autonomic Dysfunction in Autism Spectrum Disorders (ASD) (P5. 117). AAN Enterprises (2016).

[37] Bricout V-A Pace M Dumortier L Favre-Juvin A Guinot M. Autonomic responses to head-up tilt test in children with autism spectrum disorders. J Abnorm Child Psychol. (2018) 46:1121–8. 10.1007/s10802-017-0339-9 28795253

[38] Baeza-Velasco C Cohen D Hamonet C Vlamynck E Diaz L Cravero C. Autism, joint hypermobility-related disorders and pain. Front Psychiatry. (2018) 9:1–8. 10.3389/fpsyt.2018.00656 30581396 PMC6292952

[39] Casanova E Sharp J Edelson S Kelly D Casanova M. A cohort study comparing women with Autism Spectrum Disorder with and without generalized joint hypermobility. Behav Sci. (2018) 8:35. 10.3390/bs8030035 29562607 PMC5867488

[40] Asztély K Kopp S Gillberg C Waern M Bergman S. Chronic pain and health-related quality of life in women with autism and/or ADHD: a prospective longitudinal study. J Pain Res. (2019) 12:2925–32. 10.2147/JPR.S212422 31695481 PMC6804669

[41] Moore DJ Meints SM Lazaridou A Johnson D Franceschelli O Cornelius M. The effect of induced and chronic pain on attention. J Pain. (2019) 20:1353–61. 10.1016/j.jpain.2019.05.004 31077797

[42] Kasahara S Niwa S-I Matsudaira K Sato N Oka H Yamada Y. Attention-deficit/hyperactivity disorder and chronic pain. Psychosom Med. (2020) 82:346–7. 10.1097/PSY.0000000000000789 32251099

[43] Iodice V. The Postural TACHYCARDIA syndrome: A Multi-system Condition. Clinical Features, Pathophysiology, Genetics and Novel Treatment. PhD, Imperial College London (2013).

[44] Beighton P Solomon L Soskolne C. Articular mobility in an African population. Ann Rheum Dis. (1973) 32:413. 10.1136/ard.32.5.413 4751776 PMC1006136

[45] Allen M Poggiali D Whitaker K Marshall TR Kievit RA. Raincloud plots: a multi-platform tool for robust data visualization. Wellcome Open Res. (2019) 4:63. 10.12688/wellcomeopenres.15191.1 31069261 PMC6480976

[46] R Core Team,. R: A Language environment for statistical computing. R Foundation for Statistical Computing. Vienna (2020). Available online at: https://www.R-project.org/ (accessed 04.07.2021).

[47] Hayes AF. Introduction to Mediation, Moderation, and Conditional Process Analysis: A Regression-Based Approach. New York, NY: Guilford publications (2017).

[48] Eccles JA Beacher FD Gray MA Jones CL Minati L Harrison NA. Brain structure and joint hypermobility: relevance to the expression of psychiatric symptoms. Br J Psychiatry. (2012) 200:508–9. 10.1192/bjp.bp.111.092460 22539777 PMC3365276

[49] Bowen J Fatjó J Serpell JA Bulbena-Cabré A Leighton E Bulbena A. First evidence for an association between joint hypermobility and excitability in a non-human species, the domestic dog. Sci Rep. (2019) 9:8629. 10.1038/s41598-019-45096-0 31197220 PMC6565730

[50] Craig AD. How do you feel? Interoception: the sense of the physiological condition of the body. Nat Rev Neurosci. (2002) 3:655–66. 10.1038/nrn894 12154366

[51] Quigley KS Kanoski S Grill WM Barrett LF Tsakiris M. Functions of interoception: from energy regulation to experience of the self. Trends Neurosci. (2021) 44:29–38. 10.1016/j.tins.2020.09.008 33378654 PMC7780233

[52] Bonaz B Lane RD Oshinsky ML Kenny PJ Sinha R Mayer EA. Diseases, disorders, and comorbidities of interoception. Trends Neurosci. (2021) 44:39–51. 10.1016/j.tins.2020.09.009 33378656

[53] Mallorqui-Bague N Garfinkel SN Engels M Eccles JA Pailhez G Bulbena A. Neuroimaging and psychophysiological investigation of the link between anxiety, enhanced affective reactivity and interoception in people with joint hypermobility. Front Psychol. (2014) 5:1162. 10.3389/fpsyg.2014.01162 25352818 PMC4196473

[54] Rae CL Polyanska L Gould Van Praag CD Parkinson J Bouyagoub S Nagai Y. Face perception enhances insula and motor network reactivity in Tourette syndrome. Brain. (2018) 141:3249–61. 10.1093/brain/awy254 30346484 PMC6202569

[55] Rae CL Critchley HD Seth AK. A Bayesian account of the sensory-motor interactions underlying symptoms of Tourette Syndrome. Front Psychiatry. (2019) 10:29. 10.3389/fpsyt.2019.00029 30890965 PMC6412155

[56] Cleeland C. Research in cancer pain. What we know and what we need to know. Cancer. (1991) 67:823–7. 1986852 10.1002/1097-0142(19910201)67:3+<823::aid-cncr2820671412>3.0.co;2-s

[57] Kemani MK Zetterqvist V Kanstrup M Holmström L Wicksell RK. A validation of the pain interferenceindex in adults with long-standing pain. Acta Anaesthesiol Scand. (2016) 60:250–8. 10.1111/aas.12599 26310686

[58] Malek S Reinhold EJ Pearce GS. The Beighton Score as a measure of generalised joint hypermobility. Rheumatol Int. (2021) 41:1707–16. 10.1007/s00296-021-04832-4 33738549 PMC8390395

[59] Mathias CJ Low DA Iodice V Owens AP Kirbis M Grahame R. Postural tachycardia syndrome–current experience and concepts. Nat Rev Neurol. (2011) 8:22–34. 10.1038/nrneurol.2011.187 22143364

[60] Garfinkel SN Tiley C O'keeffe S Harrison NA Seth AK Critchley HD. Discrepancies between dimensions of interoception in autism: Implications for emotion and anxiety. Biol Psychol. (2016) 114:117–26. 10.1016/j.biopsycho.2015.12.003 26724504

[61] Eccles JA Thompson B Themelis K Amato M Stocks R Pound A. Beyond bones - the relevance of variants of connective tissue (hypermobility) to fibromyalgia, ME/CFS and controversies surrounding diagnostic classification: an observational study. medRxiv. (2020). 2020.02.21.20025072. 10.1101/2020.02.21.20025072 PMC7850199 33479068

