# Supplementary material for: Joint Hypermobility Links Neurodivergence to Dysautonomia and Pain
Source: Front Psychiatry. 2022 Feb 2;12:786916. doi: 10.3389/fpsyt.2021.786916 (PMC8847158; doi:10.3389/fpsyt.2021.786916)
Supplement: Supplementary Table 1 — Recorded diagnoses in neurodivergent participants. [file Table_1.docx]

Supplementary Table 1

Diagnosed neurodevelopmental and mental health conditions in neurodivergent (neurodevelopmental condition) participants

|  | Neurodevelopmental condition diagnoses | | | | Totals (including those with co-occurring neurodevelopmental condition diagnoses) | |  |
| --- | --- | --- | --- | --- | --- | --- | --- |
| Mental health diagnoses | ADHD | Autism | Tourette syndrome |  | |  | |
| Anxiety | 5 | 2 | 5 | 16 | | | |

| Depression | 5 | 2 | 0 | 8 |
| --- | --- | --- | --- | --- |

| Anxiety and depression | 2 | 1 | 3 | 6 |
| --- | --- | --- | --- | --- |
| Bipolar affective disorder and anxiety | 1 | 0 | 0 | 1 |
| Personality Disorder | 1 | 1 | 0 | 2 |
| OCD | 1 | 0 | 9 | 10 |

| None | 41 | 21 | 7 | 75 |
| --- | --- | --- | --- | --- |
